# Modeling intracellular silent oscillations and rhythmic discharge in olfactory bulb mitral cells

**DOI:** 10.1186/1471-2202-14-S1-P8

**Published:** 2013-07-08

**Authors:** Nicolas Fourcaud-Trocmé, Virginie Briffaud, Corine Amat

**Affiliations:** 1 INSERM, U1028; CNRS, UMR5292; Lyon Neuroscience Research Center, "Olfaction: from coding to memory" Team, Lyon, F-69000, France; 2 University Lyon 1, Villeurbanne, F-69000, France

## 

In mammals, the main olfactory bulb (MOB) is driven by air flow respiratory input into the nose. This slow (2-10 Hz) rhythmic input can be observed both at the local field potential (LFP) level, single cell discharge pattern level and intracellular slow oscillations of mitral cells (principal cells of the MOB). Using intracellular recordings in freely breathing anesthetized rats, we examined membrane potential slow oscillations (MPSO) in mitral cells and their relationship with their discharge patterns [1]. Interestingly, we found cells which showed no MSPO at resting membrane potential but had nevertheless a respiratory-synchronous discharge. Changing the excitability level of these cells with hyperpolarizing current injection favored the apparition of an MPSO while decreasing the cell discharge. We called such a dynamic *silent MPSO*. In this study, we use a modeling approach to show how such a silent MPSO can exist in mitral cells and favor the synchronization of cell discharge to the MOB slow rhythmic input.

First, using a simple integrate-and-fire (IF) model, we showed that the presence of strong and balanced excitatory and inhibitory inputs with similar oscillatory dynamics led the model to display a silent MPSO. This silent MPSO is characterized by a flat membrane potential in spite of strong synaptic conductance oscillations. Second, assuming that the slow oscillatory input could be decomposed as a mean input and stochastic fluctuation with oscillating amplitudes, we showed that the model could display a slow rhythmic discharge similar to our experimental observations.

However, MOB mitral cells have a complex anatomy with (i) an apical dendrite and a tuft receiving both excitatory nose input and local inhibitory regulation, and (ii) long lateral dendrites which receive a large recurrent inhibitory input from the MOB network. We thus wondered how our previous modeling results could generalize in this case. To this aim, we used a previously developed detailed mitral cell model and showed with numerical simulations that silent MPSO could be generated in this model. However, the precise dynamics of the oscillations depended on the main inhibitory input of the mitral cell. If most of the inhibition comes from the tuft, then the silent MPSO can quite easily be generated in the cell, and partial imprecision in the excitation-inhibition balance can be hindered by the apical dendrite filtering. However if a fraction of the inhibition is coming from the lateral dendrites, the silent MPSOs are more difficult to stabilize and the resulting MPSOs have complex shapes resembling some of our intracellular recordings.

Overall, we found that mitral cell silent MPSOs with respiratory-patterned discharge, that we observed experimentally, can indeed be a consequence of the precise balance of their slow oscillatory excitatory and inhibitory inputs.
